# The effect of cobalt/copper ions on the structural, thermal, optical, and emission properties of erbium zinc lead borate glasses

**DOI:** 10.1038/s41598-023-39256-6

**Published:** 2023-07-28

**Authors:** Eman O. Taha, Aly Saeed

**Affiliations:** 1 Department of Petroleum Applications, Egyptian Petroleum Research Institute (EPRI), Cairo, Egypt; 2 Mathematical and Natural Science Department, Faculty of Engineering, Egyptian Russian University, Cairo, Egypt

**Keywords:** Condensed-matter physics, Materials for optics

## Abstract

A host glass network of 70B_2_O_3_–10Pb_3_O_4_–18ZnO–2Er_2_O_3_ (ErCoCu1) was proposed and the impact of 1 mol% of Co or/and Cu ions on its structural, thermal, optical, and green emission properties was studied extensively. The X-ray diffraction spectra confirmed the amorphous structure of the produced glasses. Density and density-based parameters behavior showed that the Co or/and Cu ions fill the interstitial positions of the proposed ErCoCu1 network, causing its compactness. Both ATR-FTIR and Raman Spectra affirmed the formation of the fundamental structural units of the borate network, B–O–B linkage, BO_3_, and BO_4_. Additionally, the penetration of Co or/and Cu ions inside the ErCoCu1 converts the tetrahedral BO_4_ units to triangle BO_3_ causing its richness by non-bridging oxygens. The addition of Co or/and Cu reduces the glass transition temperature as a result of the conversion of the BO_4_ to BO_3_ units. Optical absorption spectra for the host glass ErCoCu1 showed many of the distinguished absorption bands of the Er^3+^ ion. Penetration of Co ion generates two broadbands referring to the presence of Co^2+^ ions in both tetrahedral and octahedral coordination and Co^3+^ ions in the tetrahedral coordination. In the Cu-doped glasses, the characteristic absorption bands of Cu^2+^ and Cu^+^ were observed. A green emission was generated from the ErCoCu1 glass under 380 nm excitation wavelength. Moreover, no significant effect of Co or/and Cu on the emission spectra was recorded. The considered glasses had appropriate properties qualifying them for optoelectronics and nonlinear optics applications.

## Introduction

The multiple oxidation states of the transition metal ions TMIs rich the glasses networks by many optical, electrical, and magnetic properties^1–3^. Optically, the TMIs give various specular colors to the glass networks, making them have a high optical absorption ability in the different regions of the electromagnetic spectrum such as UV, visible, and IR regions^4–6^. From the photoluminescence point of view, TMIs generate broad emission bands that have an adjustable wavelength and appropriate quantum yield^7,8^. Electrically and magnetically, the multiple oxidation states of the TMIs bring substantial modifications in the glass networks structural units by influencing the charge degree of freedom and spin, which in turn directly affect the conduction process and the electrical and magnetic nature of the glass network^9,10^. Hence, glass-containing TMIs have significant applications in photonics, electronic, optoelectronics, and magnetic domains such as light emitting diodes, optical filters, solid-state lasers, memory-switching electronics, superionic batteries, catalysis, smart electronic devices, and magnetic information storage^11–13^. Cobalt (Co^2+^/Co^3+^) and copper (Cu^+^/Cu^2+^) ions are of the most distinctive transition metal ions in enhancing the properties of various glass networks. The formation of the mixed valence states of cobalt ions (Co^2+^/Co^3+^) in octahedral (oh) and tetrahedral (Td) geometric forms inside the glass network makes it a favorable material in solar selective absorbers, fuel cells, visible and NIR-lasing materials, supercapacitors, gas sensors, and lithium-ion batteries. Cobalt imparts a blue or pink color to the glass depending on the Co^2+^ ion geometrical shape coordination (tetrahedral or octahedral) ^14–16^. Adding Cu ions to glasses networks generates two valence states, Cu^+^ and Cu^2+^, during the preparation process under normal conditions. Cu ions usually add a blue or green color to the glass network. In general, the formation of divalent copper ion Cu^2+^ can be determined based on the formed color in the glass. In addition, the Cu^2+^ ion form a broad absorption band in the visible-near infrared range that usually arises due to the octahedral coordination of Cu^2+^, while the cuprous (monovalent copper) ion Cu^+^ has a distinct absorption band in the UV region. These absorption bands are usually used to detect the presence of Cu^+^ and Cu^2+^ within the glass network^1,3,4,7^. Rare earth ions RE^3+^ possess unique properties, the foremost of which is the property of photoluminescence, which made them dominant in many photonics and optoelectronics applications^17,18^. Er^3+^ ion is among the rare earth ions that is characterized by its richness in energy levels, which made it a unique light emitter for various spectrum regions such as blue, green, red, and white light^17,18^. Borate glass is one of the most common glasses networks due to its high transparency and high thermal stability, in addition to its low melting point, which makes the ease its fabrication process. However, due to its high phonon energy, which affects negatively the quantum yield of the photoluminescence, borate glass is reinforced with heavy metal oxides such as PbO and Bi_2_O_3_^19,20^. On the other hand, the addition of PbO enhances the mechanical, thermal, and optical properties of the borate glass network^19,20^. Generally, the borate glass network, especially those reinforced with heavy metal ions is a unique host for all glass additives such as alkali ions (Li^+^, Na^+^, etc.), alkaline earth ions (Sr^2+^, Ba^2+^, etc.), transition metal ions (Zn^2+^, Co^2+^/Co^3+^, Cu^+^/Cu^2+^, etc.), post-transition metal ions (Al^3+^, Bi^3+^, etc.), and rare-earth ions (Er^3+^, Yb^3+^, etc.)^21,22^. In view of its aforementioned unique features and the multiplicity of properties that they give to glass networks depending on the concentration, type of glass network, and method of preparation, the studies continue to explore the effective role of transition metal ions in enhancing the glass properties to improve its technological performance in various fields. In 2023, O.I. Sallam et al. studied the impact of four of transition metal ions (CuO, CoO, Fe_2_O_3_, and NiO) on the photoluminescence (PL) and dielectric properties of the 20NaF–60P_2_O_5_–20Na_2_O. The authors found that adding CuO and Fe_2_O_3_ improves the dielectric parameters of their considered glass, while CoO and NiO reduce the ac conductivity. Their base glass generates emission bands at 480 and 530 nm through pumped them by excitation wavelength of 457 nm. The emission bands position and intensity strongly depended on the type of transition metal dopant^1^. Kun Lei et al. prepared in 2023 the base glass Na_2_O–B_2_O_3_–SiO_2_ by ion exchange and studied the influence of Cu^+^ ions on its structural and luminescence properties. A blue-green broadband centered at 468 nm was generated under 290 nm excitation wavelength and its intensity varied with increasing the ion exchange time^7^.

Hence and based on the foregoing, the host glass 70B_2_O_3_–10Pb_3_O_4_–18ZnO–2Er_2_O_3_ was inlaid by 1 mol% of Co or/and Cu ions (as an additive not by replacement). The structural changes as a result of the variation of Co or/and Cu ions were studied through X-ray diffraction (XRD) spectra, density and density-based parameters, Attenuated Total Reflectance-Fourier Transform Infrared (ATR-FTIR) spectra, and Raman spectra. Thermally, the glass transition temperature was measured using the Differential Scanning Calorimeter (DSC). In the optical absorption region 200–1600 nm, the optical properties of the considered glasses were studied. Finally; under the influence of the 380 nm excitation wavelength, the photoluminescence spectra in the spectral region of 380–800 nm were recorded.

## Experimental and theoretical aspects

### Materials preparation

A host glass network of a chemical composition of 70B_2_O_3_–10Pb_3_O_4_–18ZnO–2Er_2_O_3_ was prepared by the melt/annealing method. After that, two of the transition metal oxides, Co_2_O_3_ and CuO, were doped (as an additive not by replacement) with different concentrations as shown in Table 1 to study their impact on the structural, thermal, optical, and photoluminescence properties. Pure raw materials of H_3_BO_3_, Pb_3_O_4_, ZnO, Er_2_O_3_, Co_2_O_3_, and CuO were mixed in a porcelain mortar and ground well to reach a uniform color homogeneous powder. Then, the glass was melted at 1100 °C for 1 h in a porcelain crucible to obtain a homogeneous and bubbles-free molten. The molten was then poured for annealing at 300 °C in a steel mold for 30 min.

**Table 1:** The chemical composition of the produced glasses.

Glass code	Chemical composition in mol%					
	B_2_O_3_	Pb_3_O_4_	ZnO	Er_2_O_3_	Co_2_O_3_	CuO
ErCoCu1	70	10	18	2	0	0
ErCoCu2	70	10	18	2	1	0
ErCoCu3	70	10	18	2	0	1
ErCoCu4	70	10	18	2	0.5	0.5
ErCoCu5	70	10	18	2	0.75	0.25
ErCoCu6	70	10	18	2	0.25	0.75

### Measurements and theoretical aspects

#### Structural properties

First, the formation of the amorphous phase of the prepared materials was tested by X-ray diffraction (XRD) patterns. A Philips X-ray diffractometer using a monochromatic Cu-Kα radiation of wavelength 1.54056 Å was used to record the X-ray diffraction spectra. Density and density-based parameters effectively explore the impact of the additives on the physical properties of glass networks, so the density $\rho$ was measured using Archimedes' principle according to Eq. 1^3,9,23,24^ then density-based parameters (molar volume ${V}_{m}$, mean boron-boron separation ${d}_{B-B}$, oxygen packing density $OPD$, and packing density $PD$) were deduced using the Eqs. 2^3,9,25–27^, 3^12,28^, 4^12,26^, and 5^26^.

1 $$\rho =\frac{{W}_{a}}{{W}_{a}-{W}_{l}}\times {\rho }_{l}$$

2 $${V}_{m}=\frac{M}{\rho }$$

3 $${d}_{B-B}={\left(\frac{{V}_{m}^{B}}{{N}_{A}}\right)}^{1/3}$$

4 $$OPD=\frac{\rho }{M}\times n$$

5 $$PD=\frac{\rho }{M}\sum_{i}{x}_{i}{V}_{i}$$

where ${W}_{a}$ & ${W}_{l}$, ${\rho }_{l}$, $M$, ${N}_{A}$, ${V}_{m}^{B}$, $n$, ${x}_{i}$, and ${V}_{i}$ are weight of the sample in air & liquid, the liquid density, the molar mass of the glass sample, the Avogadro’s number, the volume containing 1 mol of boron ions inside the studied glass network, the oxygen atoms number per formula unit, the mole fraction, and the packing factor

The values of the volume containing one mol of boron ${V}_{m}^{B}$ and packing factor ${V}_{i}$ of the ith oxide with a chemical formula ${A}_{b}{O}_{c}$ were calculated using

$${V}_{m}^{B}=\frac{{V}_{m}}{2\left(1-{X}_{B}\right)}$$

$${V}_{i}=\frac{4\pi {N}_{A}}{3}\left(b{r}_{A}^{3}+c{r}_{B}^{3}\right)$$

where ${X}_{B}$, and ${r}_{A}$ & ${r}_{B}$ are the molar fraction of B_2_O_3_ and the ionic radii of the cation & the anion.

Attenuated Total Reflection-Fourier Transform Infrared Spectroscopy (ATR-FTIR), (Alpha-Bruker) was used to study the functional groups of the studied glasses in the spectral range of 400–4000 cm^−1^. Gaussian deconvolution was conducted to pry the origin of the formed broadbands in the ATR-FTIR spectra. The formed tetrahedral BO_4_ (${N}_{4}$) and trigonal BO_3_ (${N}_{3}$) units ratios inside the considered glass network were calculated using Eqs. 6 and 7^26^.

6 $${N}_{4}=\frac{Area \left(B{O}_{4}\right)}{Area \left(B{O}_{3}\right)+Area \left(B{O}_{4}\right)}$$

7 $${N}_{3}=\frac{Area \left(B{O}_{3}\right)}{Area \left(B{O}_{3}\right)+Area \left(B{O}_{4}\right)}$$

Raman spectra were recorded for the considered glasses by SENTERRA Dispersive Raman Microscope (Bruker) equipped with a diode Nd:YAG laser in the spectral region of 500–4000 cm^−1^. A narrow-spectrum 532 nm laser excitation system was used to illuminate the produced glasses. Also as the same as of ATR-FTIR, a Gaussian deconvolution to resolve the formed broadbands in the Raman spectra was carried out.

#### Thermal properties

The glass transition temperature of the considered glasses was deduced through a Differential Scanning Calorimeter test using a Mettler–Toledo Instruments, at a 5 °C/min heating rate.

#### Optical properties

UV–VIS-NIR absorption spectra were measured using a JASCO V-670 UV/Vis spectrophotometer in the spectral region of 200–1600 nm with a 2 nm resolution. Smooth and flat glass samples with dimensions 2 cm^2^ and thicknesses 1.1–1.4 mm were used in the optical spectra measurements. To determine the existence of various oxidation states of both Co and Cu ions in different geometrical shapes (octahedral and tetrahedral coordination), a deconvolution of the optical absorption spectra was conducted. Based on the obtained absorption bands of Co ions, the ligand field parameters around Co ion; crystal field splitting coefficient 10Dq, Racah parameters B&C, and nephelauxetic ratio β were estimated through the following relations^24,25,29.^

8 $$B=\frac{1}{510}\left[7\left({\nu }_{2}+{\nu }_{3}\right)\pm {\left\{49{\left({\nu }_{2}+{\nu }_{3}\right)}^{2}+680{\left({\nu }_{2}-{\nu }_{3}\right)}^{2}\right\}}^\frac{1}{2}\right]$$

9 $$10Dq=\frac{1}{3}\left({\nu }_{2}+{\nu }_{3}\right)-5B$$

10 $$C=4.63B$$

11 $$\beta =\frac{{B}_{complex}}{{B}_{free ion}}$$

where ${B}_{free ion}$ of Co is 1120 and ${\nu }_{2}$ and ${\nu }_{3}$ pointed to the bands corresponding to the energy of electronic transitions in tetrahedral Co^2+^ ions, ${\nu }_{2}$ is the electronic transition corresponding to the visible region and ${\nu }_{3}$ is that to the NIR region.

Based on the optical absorption spectra, the optical band gap of the considered glasses was deduced according to Mott-Davis theory using the following equation^9,10,14,24^.

12 $$ \alpha h\nu  = B\left( {h\nu  - E_{{opt}} } \right)^{n}  $$

where $\alpha$, $h\nu$, $B$, ${E}_{opt}$ are the absorption coefficient, the incident photon energy, the band tailing parameter, and the optical band gap energy respectively. The $n$ signifies the kind of the occurred electronic transition and take values 1/3 & 1/2 for direct transitions and 2 & 3 for indirect ones. Generally, for glassy materials, the indirect allowed transition is the dominant; hence, the ${E}_{opt}$ values were deduced for the $n=2$ only. The value of ${E}_{opt}$ estimated through plot the Tauc relation between $h\nu$ and ${(\alpha h\nu )}^{0.5}$ and by extrapolating the linear part of the curve at ${(\alpha h\nu )}^{0.5}=0$.

To measure the disorder degree in the considered glasses, the band tail, which known as Urbach energy $\Delta E$ was estimated through the following equation^9,10,14,24.^

13 $$ \alpha  = \beta e^{{h\nu/{\Delta}E}}  $$

where β is a constant.

The value of Urbach energy determine through the inclination of the linear regions of $ln\alpha -h\nu$ relation and taking its reciprocal.

The steepness coefficient *S*, which measures the width of the band-tail in the main gap, was calculated using the formula^28,29.^

14 $$\alpha =\beta {e}^{\left[\frac{S\left(h\nu -{E}_{g}\right)}{{K}_{B}T}\right]}$$

where $\beta$, ${K}_{B}$, and $T$ are the exponential constant, Boltzmann constant, and room temperature.

Based on some mathematical contractions, the values of the steepness coefficient can be obtained through a simple relationship that links the steepness coefficient with Urbach energy, which is

15 $$S=\frac{{K}_{B}T}{\Delta E}$$

Depending on the band gap values, the conducting behavior of the solid (conductor, semiconductor, or insulator) can be characterized. Metallization criterion (M), which is calculated from Eq. 16, is used to determine the conducting behavior of the solid precisely. According to Herzfeld's theory of metallization of the condensed matter, the M value may be approached to zero, close to one, or stands between them, which reflects the conductive, insulating, or semiconducting nature of the solid respectively^28,29^.

16 $$M={\left(\frac{{E}_{g}}{20}\right)}^{0.5}$$

#### Photoluminescence

Agilent—Cary eclipse fluorescence spectrophotometer equipped with Xe-lamp was used to record the emission spectra in the spectral region of 380–800 nm. The considered glasses were excited using 380 nm excitation wavelength.

## Results and discussion

### Structural properties

#### XRD

**Figure 1:**
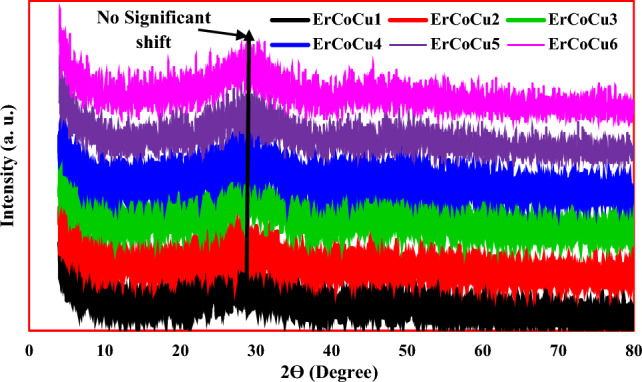
X-ray diffraction patterns of the produced ErCoCu glasses.

The X-ray diffraction spectra, which are displayed in Fig. 1 signify the amorphicity phase formation of the prepared materials, where, as observed, a broad halo in the spectral range of 22–31° has appeared. It is known that the borate glass forms a broad halo in the spectral range $2\theta =25{-}30$°^30,31^. So, the observed widening in the formed X-ray diffraction peak for the proposed host network, which extended from 22° to 31° arose as a result of the role of Pb_3_O_4_, ZnO, and Er_2_O_3_ in the network formation. No significant shift in the formed broad halo was observed with the inclusion of Co or/and Cu ions, due to they are being added in small concentrations.

#### Density and density-based parameters

Table 2 displays the variation of density and density-based parameters, molar volume ${V}_{m}$, mean boron-boron separation ${d}_{B-B}$, oxygen packing density $OPD$, and packing density $PD$, as a result of the penetration of Co or/and Cu ions. First and in general, a weak augmentation in the density and reduction in molar volume with the inlying of Co or/and Cu ions compared to the host glass ErCoCu1 was observed as listed in Table 2. The density augmentation resulted from the fact that the two suggested transition metal ions, Co and Cu, were added to the host network as a doped and not as a substitution. The observed growth in the density and the resulting reduction in molar volume arose from the filling of the interstitial spaces of the glass network by the Co or/and Cu ions, causing shrinking of the interfacial distances and tighter packing. The shrinking of the mean boron-boron separation confirmed the role of the Co or/and Cu ions in filling the interstitial spaces within the studied network, as their penetration into these voids causes them to rival the boron atoms causing their displacement towards each other. As a result of filling the interstitial spaces with Co or/and Cu ions and reducing the mean boron-boron separation, the considered glass network was tightened, which was evident in the increase in OPD and PD as listed in Table 2. On the other hand, the glass containing higher Cu ions concentrations (ErCoCu3 and ErCoCu6) than Co ions (ErCoCu2 and ErCoCu5) showed a higher density due to the higher molecular mass of Cu (63.5 gm/mol) compared to Co (58.933 g/mol). On the other hand, due to the ionic radius of Cu (0.073 nm) is higher than that of Co (0.072 nm), the range of its filling to the network voids had expanded causing a higher reduction in the molar volume for the glass containing higher concentrations of Cu compared to that containing higher concentrations of Co.

**Table 2:** Variation of the structural parameters with the inclusion of Co or/and Cu ions.

Glass code	$\rho$ gm/cm^3^	${V}_{m}$ cm^3^/mol	${d}_{B-B}$ Å	OPD	PD	N_4_	N_3_
ErCoCu1	3.621	38.548	4.743	73.305	0.404	0.463	0.537
ErCoCu2	3.685	38.327	4.734	74.253	0.413	0.436	0.564
ErCoCu3	3.697	37.973	4.719	74.392	0.412	0.426	0.574
ErCoCu4	3.691	38.152	4.727	74.323	0.413	0.425	0.575
ErCoCu5	3.678	38.344	4.734	74.288	0.411	0.405	0.595
ErCoCu6	3.684	38.166	4.727	74.358	0.411	0.413	0.587

#### ATR-FTIR

**Figure 2:**
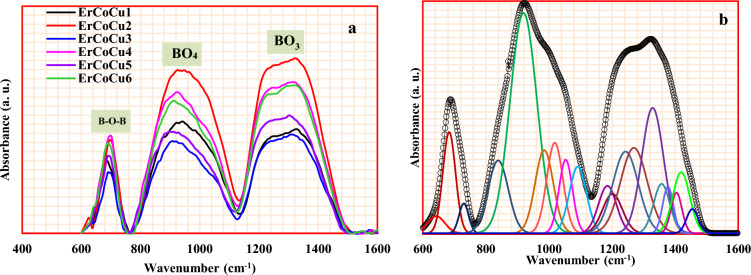
(**a**) ATR-FTIR spectra of the considered ErCoCu1-6 glasses and (**b**) The deconvoluted spectra of ATR-FTIR of ErCoCu1 glass as an example.

The main building block units of the borate network, B–O–B linkage, BO_4_ units, and BO_3_ units were observed in the FTIR spectra as shown in Fig. 2a. Some modifications in the intensity and position of the absorption bands with the penetration of Co or/and Cu ions to the host glass network were observed. The Gaussian deconvolution, host glass ErCoCu1 as an example, as shown in Fig. 2b shows the origin of the overlapped bands, which formed the broadbands in the FTIR spectra and examines the occurred structural modifications within the network as a result of Co or/and Cu inclusion.

In the host glass ErCoCu1, nineteen primary bands have appeared at 643, 684, 733, 839, 919, 984, 1018, 1053, 1091, 1185, 1201, 1243, 1269, 1329, 1357, 1377, 1404, 1419, and 1455 cm^−1^. The characteristic bands of B–O–B bending vibrations in BO_3_ triangles, which centered at 690 cm^−1^ decomposed to 643 cm^−1^ and 684 & 733 cm^−1^. The band at 643 cm^−1^ refers to the asymmetric bond bending vibrations of BO_3_, while that are 684 and 733 cm^−1^ refers to symmetric bond bending vibrations ^28–33^. Co ions inclusion (ErCoCu2 glass) increased the intensity and relative area of this band, while an opposite trend was observed with Cu ions inclusion (ErCoCu3). This behavior reflects the network richness of the glass containing 1 mol% of Co ions by B–O–B bending vibration compared to that containing 1 mol% of Cu ions. In the glass containing the mixture of Co and Cu ions (ErCoCu4, ErCoCu5, and ErCoCu6), it was observed that the role of Co ions in enhancing the intensity and relative area of this band is dominant. Finally, a shift towards the higher energy occurred in the glass containing 0.5 mol% of Co and 0.5 mol% of Cu while the relative area increased. The deconvolution of BO_4_ unit broadband, which appeared here in the spectral region 770–1120 cm^−1^ emerged six absorption bands at 839, 919, 984, 1018, 1053, and 1091 cm^−1^. The located band at 839 cm^−1^ attributed to the B–O stretching vibration of NBOs in BO_4_ units (${\mathrm{NBO}}_{{\mathbf{B}\mathbf{O}}_{4}}$)^28,29,34,35^. This band also may have arisen as a result of the Pb–O bond vibration of PbO_n_ pyramidal units^36^. The bands at 919, 984, 1018, 1053, and 1091 cm^−1^ refer to the B–O stretching vibrations units in different structural groups; di, tri, meta, pyro, and ortho borate chains ^35–37^. Finally, ten bands arose as a result of resolving the BO_3_ broadband in the spectral range of 1125–1500 cm^−1^. The B–O asymmetric stretching vibrations of NBO of trigonal atoms (${\mathrm{NBO}}_{{\mathbf{B}\mathbf{O}}_{3}}$) was observed at 1243 cm^−1^^36–39^. The rest of the ten bands, which appeared at 1185, 1201, 1269, 1329, 1357, 1377, 1404, 1419, and 1455 cm^−1^ arose due to the symmetric and asymmetric of B–O stretching vibrations of trigonal [BO_3_]^3−^ units in various structural groups; di, tri, meta, pyro, and ortho borate chains^28,29,36–41^. Generally, the inclusion of Co or/and Cu ions to the considered glass network caused a reduction in intensity and relative area of the 839 cm^−1^ (${\mathrm{NBO}}_{{\mathbf{B}\mathbf{O}}_{4}}$ band), while an augmentation in both of them was observed for the 1243 cm^-1^ band (${\mathrm{NBO}}_{{\mathbf{B}\mathbf{O}}_{3}}$ band) refereeing to the conversion of BO_4_ units to BO_3_ ones. The penetration of Co or/and Cu ions led to the enrichment of the glass network with non-bridging oxygens (NBOs) units compared to the bridging (BOs) ones, which is evident from the behavior of N_3_ and N_4_ listed in Table 2.

#### Raman spectroscopy

**Figure 3:**
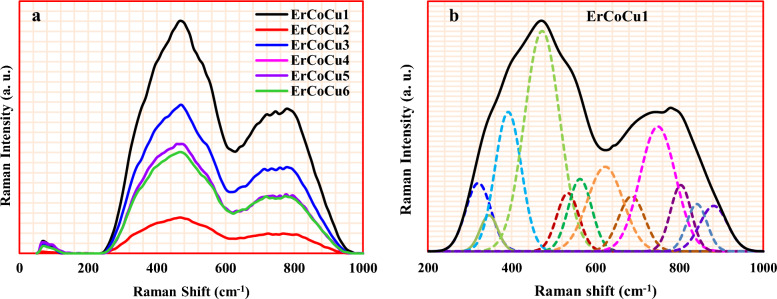
(**a**) Raman spectra of the considered ErCoCu1-6 glasses and (**b**) The deconvoluted spectra (host glass ErCoCu1 as an example).

Figure 3 shows the recorded Raman spectra for the considered glasses and their deconvolutions (host glass ErCoCu1 as an example). In Fig. 3a, the observed band in the low energy region 50–125 cm^−1^ arose due to the vibrational modes of BO_3_ and BO_4_ units^41^. In the deconvolution of host glass ErCoCu1 (in the spectra range 200–1000 cm^−1^), twelve bands are appeared at 320, 345, 391, 473, 534, 563, 623, 686, 749, 803, 842, and 882 cm^−1^ as shown in Fig. 3b. The lower frequency bands 320, 345, and 391 cm^−1^ usually refers to the rotational and vibrational modes of metal–oxygen, here 320 and 345 cm^−1^ arose as a result of the stretching vibrations and bending mode of Zn–O in ZnO_4_ structural units^42,43^, while that at 391 cm^−1^ due to Pb–O bonds in PbO_3_ structural units^44^. The isolated diborate groups of the considered borate network appeared at 473 cm^−1^^45^. The centered band at 534 cm^−1^ arose as a result of the deformation mode of B–O–B linkage^41^, while that appeared at 563 cm^−1^ arose due to the destruction of diborate groups and the formation of “'loose” BO_4_ˉ units^46^. The band at 623 cm^−1^ arose due to the symmetric stretching of metaborate rings, while that at 686 cm^−1^ assigned to pentaborate groups^45–50^. The centered band at 623 cm^−1^ also signifies the formation of a bending mode of the Pb–O–B links^45^. The appeared band at 749 cm^−1^ arose as a result of the symmetric breathing vibrations of six-member rings with one or two BO_4_ tetrahedra^48,49^. The boroxol ring breathing of the considered borate network appeared at 803 cm^−1^^47,51,52^. The pyroborate vibrations and ortho-borate groups arose at 842 and 882 cm^−1^ respectively^50,53^.

### Thermal properties

**Figure 4:**
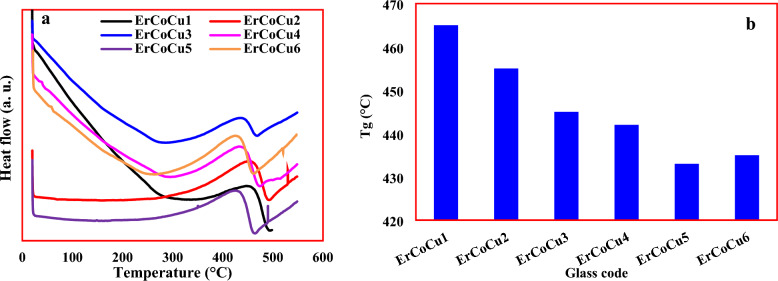
(**a**) DSC profile of the considered glasses and (**b**) Variation of the glass transition temperature with Co or/and Cu ions.

The thermal profile of the considered glasses and the estimated glass transition temperatures ${T}_{g}$ are shown in Fig. 4a,b. As shown in Fig. 4b, the glass transition temperature of the host glass ErCoCu1 appeared at 465 C. With the penetration of Co or/and Cu ions into the considered host glass network, the glass transition temperatures shrunk as shown in Fig. 4b. The observed reduction in the ${T}_{g}$ arose due to the conversion of the higher bond dissociation energy BO_4_ (62.8–82.2 kJ/cm^3^) to the lower one BO_3_ (15.6–16.4 kJ/cm^3^)^54,55^, which is clearly shown in Table 2 in the behavior of N_3_ and N_4_. The occurred contraction in the ${T}_{g}$ was entirely in tune with the concentration of BO_3_ within the glass network, where the glasses containing higher concentrations of BO_3_ showed a lower glass transition temperature than those containing lower concentrations.

### Optical properties

**Figure 5:**
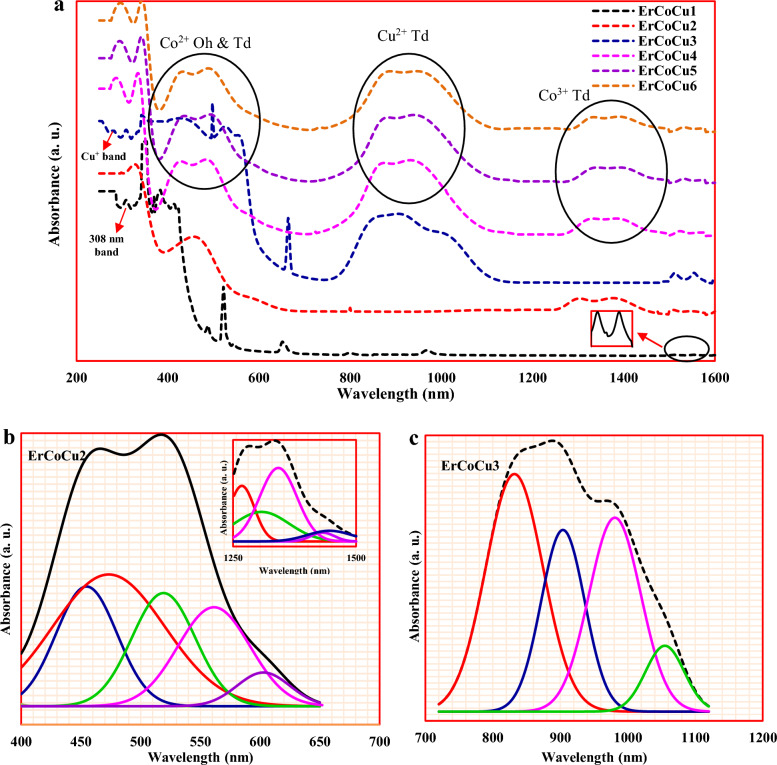
(**a**) The obtained optical absorption spectra of the considered glasses and the deconclusion of (**b**) ErCoCu2 and (**c**) ErCoCu3 glasses.

In the host glass ErCoCu1, a band at 308 nm and ten of the characteristic absorption bands of Er^3+^ ion at 346, 384, 426, 522, 546, 650, 800, 980, 1512, and 1554 nm have appeared as displayed in Fig. 5a. The absorption band located at 308 nm arose due to of the electron transition in the non-bridging oxygen and/or the electron transition in the divalent Pb^2+^ ions^56,57^. The centered bands at 346, 384, 426, 522, 546, 650, 800, 980, and 1554 nm are attributed to the occurred transitions in Er^3+^ ion between the ground state ^4^I_15/2_ and the excited states ^2^K_15/2_, ^4^G_11/2_, ^4^F_3/2_, ^2^H_11/2_, ^4^S_3/2_,^4^F_9/2_, ^4^I_9/2_, ^4^I_11/2_, and ^4^I_13/2_ respectively^58–61^. The observed band at 1512 nm arose as a result of the split ^4^I_13/2_ level^59,60^. In Co doped glass ErCoCu2 as shown in Fig. 5a, two broadbands appeared in the spectral region of 400–650 nm and 1250–1500 nm in addition to the presence of the bands at 1512 and 1554 nm, which results from ^4^I_15/2_ $\to$ ^4^I_13/2_ transition as mentioned previously^59,61^. The deconvolution of the appeared two broadbands generated ten bands at 452, 472, 522, 566, 610 1272, 1310, 1346, 1432, and 1448 nm as displayed in Fig. 5b. The two bands at 452 and 522 nm arose due to the transition ^4^I_11/2_ $\to$ ^4^F_5/2_ and ^4^I_15/2_ $\to$ ^2^H_11/2_ in Er^3+^ ions^59,60^. The located bands at 472 and 566 nm arose due to the transitions ^4^T_1g_(F)$\to$ ^2^T_2g_(F) in the octahedral Co^2+^ and spin-forbidden transitions ^4^A_2g_(^4^F)$\to$ ^4^T_1g_(^4^P) in the tetrahedral Co^2+^, while that at 610 nm arose due to ^5^T_2g_ $\to$ ^5^E_g_ transition in the octahedral Co^3+^^32,62–65^. The ground state ^4^F of tetrahedral field of Co^2+^ splits to ^4^A_2_, ^4^T_2_, and ^4^T_1_, while ^2^G splits to ^2^A_1g_ (G), ^2^T_1g_(G), ^2^T_2g_(G), and ^2^E_g_(G) levels^66,67^. Moreover, ^4^P only transforms to ^4^T_1_ (^4^P) level.

So, the bands centered at 1272, 1310, 1432, and 1448 nm arose as a result to the transition between the ground state $${\Gamma }_{8}$$(^4^A_2_, ^4^F) and the excited states $${\Gamma }_{6}, {{\Gamma }_{7+8},\Gamma }_{7} \mathrm{and }{\Gamma }_{8}$$ of ^4^T_1_(^4^F)^66–70^ due to the first and second-order spin–orbit coupling effects. The absence of the three bands, which appeared at 346, 384, and 426 nm in ErCoCu1 in this sample, maybe due to their overlap with the two bands appearing at 452 and 472 nm. In Cu doped glass ErCuCo3, in addition to the bands located at 288, 314, 354, 384, 414, 492, 522, 548, 984, 1540, and 1584; a broadband appeared in the region of 740–1140 nm and deconvoluted to 838, 908, 984, and 1058 nm as shown in Fig. 5c. The copper ion usually exists in the two most stable valence states, Cu^+^ and Cu^2+^. The fulfilled 3d^10^ configuration cuprous ion Cu^+^ shows an absorption band in the UV-blue region due to the 3d^10^ → 3d^9^ 4s^1^ transition, therefore the centered band at 288 nm is refers to the existence of Cu^+^ ion in the ErCoCu3 glass^71,72^. For copper Cu^2+^, which is usually present in octahedral coordination, during the melting process; a splitting in the d-orbitals into the doubly degenerate ^2^ $${E}_{g}$$ (higher energy) and the triply degenerate ^2^ $${T}_{2g}$$ (lower energy) occurs. Moreover and due to the tetrahedral distortion, ^2^Eg splits to ^2^ $${B}_{2g}$$($${dx}^{2}-{y}^{2}$$) and ^2^ $${A}_{2g}$$($${dz}^{2}$$), while ^2^T_2g_ splits to ^2^ $${B}_{2g}$$($${d}_{xy})$$ and ^2^ $${E}_{g}$$($${d}_{xz},{d}_{yz})$$ through the Jahn–teller effect, which causes a breadth in the shape of the formed peak. Hence, the located bands at 838 (11,933 cm^−1^), 908 (11,013 cm^−1^), and 1058 nm (9452 cm^−1^) are assigned to ^2^ $${E}_{g}\to $$ ^2^ $${B}_{1g}$$, ^2^ $${B}_{2g}\to $$ ^2^ $${B}_{1g}$$, and ^2^ $${A}_{1g}\to $$ ^2^ $${B}_{1g}$$ transitions respectively^73–75^. The others appeared bands in the ErCoCu3 glass at 314, 354, 384, 414, 492, 522, 548, 984, and 1540 & 1584 nm are assigned to the transition in Er^3+^ ion from the ground state ^4^I_15/2_ to the excited states ^2^D_3/2_, ^2^K_15/2_, ^4^G_11/2_, ^4^F_3/2_, ^4^F_7/2_, ^2^H_11/2_, ^4^S_3/2_,^4^I_11/2_, and ^4^I_13/2_ respectively^58,59,76,77^. In the ErCoCu4, ErCoCu5, and ErCoCu6 glasses, which contain a mixture of Co and Cu ions, the same broadbands are formed as shown in Fig. 5a in the spectral ranges of 400–650, 740–1140, and 1250–1500 nm signifying the presence of octahedral (Oh) and tetrahedral (Td) of Co^2+^, tetrahedral of Cu^2+^, and tetrahedral Co^3+^ respectively. It is also worth mentioning that, the characteristic band of Cu^+^ ions is continued to exist in these glasses.

The deduced values of the crystal field splitting coefficient 10Dq, Racah parameters B&C, and nephelauxetic ratio β are listed in Table 3. It was found that the Racah parameters, which generally use to measure the Coulomb repulsion within the d-shell decrease with the increase of Cu ions and a decrease of Co ions concentrations. The observed reduction in Rach parameters refers to the covalency nature of the bonds between Co ions and ligands. On the other hand, the observed reduction in the 10Dq signifies that the Co^2+^ ions have a strong localization in the considered glass network. Nephelauxetic ratio $\beta$, which measures the stability of ions (Co^2+^ ions here) complexes and their interaction mechanisms, was found to be increased with Co ions augmentation and Cu ions reduction. The reported growth in Nephelauxetic ratio values indicated the augmentation in the stability of Co^2+^ ions in the considered glasses. The 10Dq/B ratio, which measures the interaction strength, showed that the crystal field sites of the considered glasses are within the strong interaction regime and tend toward a strong crystal field.

**Table 3:** Racah parameters B&C, crystal field splitting coefficient 10Dq, Nephelauxetic ratio β, 10Dq/B, optical band gap ${\mathrm{E}}_{\mathrm{g}}$, Urbach energy $\mathrm{\Delta E}$, steepness coefficient S, metallization criterion M, and non-linear refractive index ${\mathrm{n}}_{2}$.

Glass code	B	C	10Dq	$$\beta $$	10Dq/B	$${E}_{g}$$(eV)	$$\Delta E$$(eV)	S	M	$${\mathrm{n}}_{2}$$ ($${10}^{-11})$$
ErCoCu1	–	–	–	–	–	2.425	0.476	0.053	0.348	3.644
ErCoCu2	961.609	4452.250	3710.048	0.859	3.858	2.211	0.826	0.031	0.332	5.272
ErCoCu3	–	–	–	–	–	1.624	0.794	0.032	0.285	18.115
ErCoCu4	932.308	4316.585	3670.004	0.832	3.936	2.189	0.556	0.045	0.331	5.488
ErCoCu5	937.334	3684.762	4339.857	0.837	3.931	2.210	0.833	0.030	0.332	5.282
ErCoCu6	920.588	4262.324	3640.348	0.822	3.954	2.233	0.870	0.029	0.334	5.068

**Figure 6:**
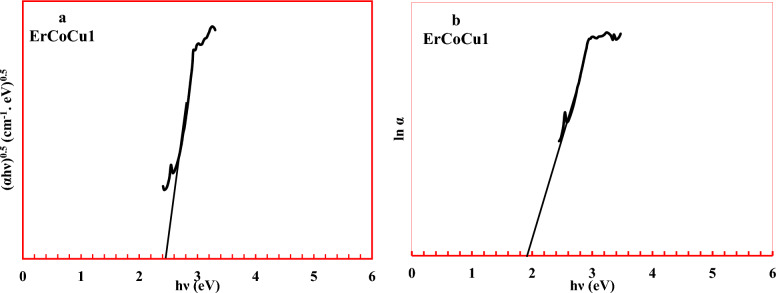
(**a**) Tauc plot and (**b**) $h\nu$ vs. ${ln}\alpha$ relation.

Tauc relationship between $h\nu$ and ${(\alpha h\nu )}^{0.5}$ was plotted as shown in Fig. 6a (glass ErCoCu1 as an example) to deduce the values of optical band gap for the considered glass. On the other hand, to estimate the Urbach energy values, a relationship between $h\nu$ and $\mathrm{ln}\alpha$ was plotted as shown in Fig. 6b (glass ErCoCu1 as an example). Generally, a reduction in optical band gap and the augmentation in Urbach energy with the addition of Co or/and Cu ions compared to the host glass was observed as listed in Table 3.

There are two main reasons for the occurred reduction in the optical band gap and augmentation in Urbach energy. The general one is the formed non-bridged oxygen NBOs in the energy gap near valence and conduction edges. The NBOs behave like donor centers inside the band gap, which cause its shrinking. Also, the linked excited electrons by the non-bridging oxygen are less tight than those linked by the bridging oxygen, which in turn decreases the optical band gap. The specific one is that (i) the gradual augmentation of Co ions in the octahedral position formed a large number of donor centers leading to overlapping between the trapped excited states of localized electrons on Co^2+^ sites and the unfilled 3d states on the neighboring Co^3+^ sites. Hence, a wide extension of the impurity or polaron band in the band gap takes place leading to a reduction in the optical band gap^16^. (ii) Cu ions like Co create a large number of donor centers. In Cu ions, the trapped excited states of localized electrons are on Cu^+^ sites and overlap with the unfilled 3d states on the neighboring Cu^2+^ sites^78,79^. The width of the formed tails due to the agglomerations of NBO, Co, and Cu in the main band gap and the augmentation of the disorder were clearly confirmed by the obtained values of steepness coefficient S. Inclusion of Co or/and Cu reduce the value of steepness coefficient reflecting the shrinking of the edge broadening confirming the disorder augmentation and band gap reduction. The optical band gap values of the glasses containing Co or/and Cu ranged between 1.62 and 2.23 eV, which means that they have a semiconducting nature. The obtained values of the metallization criterion as listed in Table 3 confirmed the semiconducting nature of the considered glasses. The small values of the metalization criterion of the considered glasses refer that the width of both valence and conduction bands becomes large, generating a narrow band gap and enhancing the tendency of the glass for the semiconducting nature. Moreover, the values of the metallization criterion, which ranged from 0.285 to 0.348 indicated that the considered glasses have non-linear refractive indices, which means those glasses have non-linear optical properties^80^. The non-linear refractive index of the considered glasses was computed using the equation ^81,82.^

17 $${n}_{2}=\frac{B}{{E}_{g}^{4}}$$

where $$B=1.26\times {10}^{-9} e{V}^{4}$$.

Generally, compared to the host glass ErCoCu1, the glasses containing Co or/and Cu possessed high non-linear refractive indices as listed in Table 3, which arose as a result of the increased disorder within the glass network with the penetration of Co or/and Cu ions as confirmed by Urbach energy. The growth of the non-linear refractive index of the ErCoCu1 with the introduction of Co or/and Cu confirmed the enhancement of the non-linear optical properties of the produced glasses.

### Emission analysis

#### Emission spectra and electronic transition

**Figure 7:**
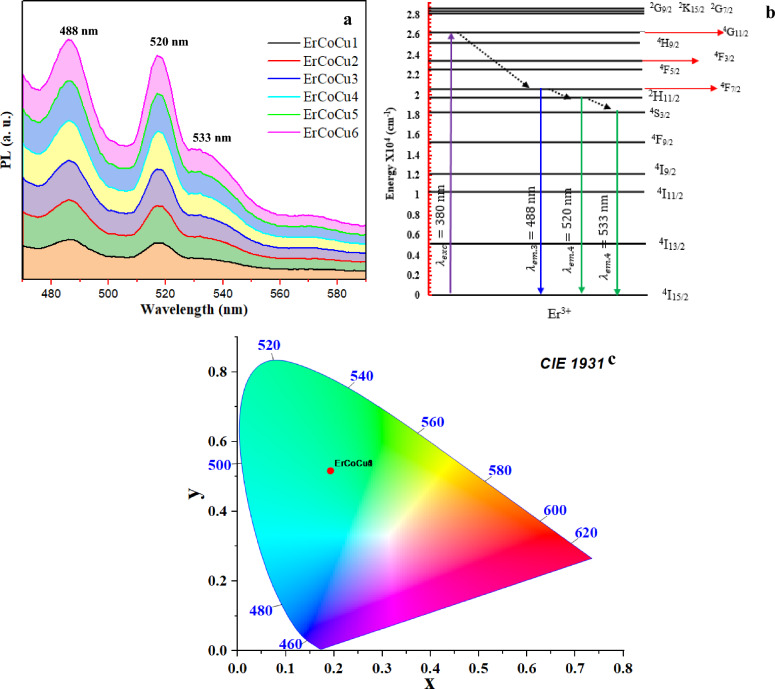
(**a**) Emission spectra of ErCoCu glass series, (**b**) The corresponding Er-electronic transition, and (**c**) The CIE1931 chromaticity diagram.

Through excitation of ErCoCu1 glass using the 380 nm, three emission bands; one in the blue light region at 488 nm and two in the green region at 520 and 533 nm are generated as shown in Fig. 7a.

Under 380 nm excitations wavelength, a quite well population of ^4^G_11/2_ occurred from the ground state absorption (GSA) ^4^I_15/2_ as shown in Fig. 7b. Non-radioactive decay via transition to the ^4^F_7/2_ excited state occurred, followed by radiative decay generating three lines of blue-green emission at 488, 520, and 533 nm. Sometimes, multi-photon electron relaxation from the excited state ^4^S_3/2_ to ^4^F_9/2_ takes place followed by the ^4^F_9/2_ $\to$ ^4^I_15/2_ transition, leading to the emission of a red wavelength line, which is not observed here. Not noticing a red emission here means that the ^4^S_3/2_ $\to$ ^4^F_9/2_ relaxation is not occurring. No variation in the observed emission bands, in position or intensity, was observed with the inclusion of Co or/and Cu. The absence of any change in the emission spectra means that the used wavelength cannot cause any excitation in the Co or Cu ions. Moreover, no energy transfer occurred between Co and Er^3+^ or Cu and Er^3+^.

#### Green light emission and colorimetric analysis

The appropriate combination of the emitted blue and green emissions generates a green light as shown in the CIE 1931 chromaticity diagram in Fig. 7c. The CIE coordinates of the considered glasses were located at ${x}_{s}=0.192$ and ${y}_{s}=0.517$, which is close to the green light of the National Television System Committee (NTSC). The absence of the effect of Co or Cu ions on the intensity and position of the emission bands was clearly reflected on the coordinates of the generated green color, as they all appeared in the same position without any significant change. Xiaojian Pan et al. obtained almost the same coordinates for the emitted green light from CaNb2O6:Tb^3+^ phosphor under 260 nm excitation^83^. The color purity of the emitted green light was calculated by the following relation^17^

18 $$P\%=\frac{\sqrt{{\left({x}_{s}-{x}_{i}\right)}^{2}+{\left({y}_{s}-{y}_{i}\right)}^{2}}}{\sqrt{{\left({x}_{d}-{x}_{i}\right)}^{2}+{\left({y}_{d}-{y}_{i}\right)}^{2}}}\times 100$$

where $\left({x}_{d}, {y}_{d}\right)$, $\left({x}_{s}, {y}_{s}\right)$, and $\left({x}_{i}, {y}_{i}\right)$ are the dominant wavelength’s color coordinates, studied samples color coordinate, and ideal white color coordinates respectively.

The dominant wavelength of the considered glasses is $\sim$ 516 nm and has coordinates (0.022, 0.780). Here, the color purity of the considered glasses is 42.579%.

## Conclusion

Impact of 1 mol% of Co or/and Cu ions on the structural, thermal, optical, and green emission of a host glass of 70B_2_O_3_–10Pb_3_O_4_–18ZnO–2Er_2_O_3_ was explored extensively. Both Co or/and Cu have an effective role in modifying the structural properties of the considered glass network by enriching it with non-bridging oxygen, and the formation of different oxidation states for Co and Cu; Co^2+^/Co^3+^ and Cu^+^/Cu^2+^. The penetration of Co and Cu ions into the studied glass network resulted in a decrease in the glass transition temperature. The optical absorption spectra indicated to the formation of tetrahedral/octahedral coordination of Co^2+^, octahedral of Co^3+^, cuprous Cu^+^, and octahedral coordination of Cu^2+^ within the considered glass network. The formed band-tail of Co and Cu ions near valence and conduction band edges reduced the optical band gap of the host glass ErCoCu1 from 2.425 to 2.211 eV, 1.624, 2.189, 2.210, and 2.233 eV for ErCoCu2, ErCoCu3, ErCoCu4, ErCoCu5, and ErCoCu6 respectively, which enhanced their semiconducting nature. Morever, the penetration of Co or/and Cu reduced the metalization criterion by 4.023–18.103%, whlie the non-liner refractive index augmentaed from $3.644\times {10}^{-11}$ for the host glass ErCoCu1 to $3.644\times {10}^{-11}$, $5.272\times {10}^{-11}$, $18.115\times {10}^{-11}$, $5.282\times {10}^{-11}$, and $5.068\times {10}^{-11}$ for ErCoCu2, ErCoCu3, ErCoCu4, ErCoCu5, and ErCoCu6 respectively referring to their sutability for non-linear optical devices. The estimated ligand field parameters referred to the strong localization of Co^2+^ ions and more covalency and stability within the glass network. A green emission with a color purity 42.579% was produced through pump the host glass by 380 nm. In contrast to the structural, thermal, and optical properties, no effect of Co and Cu ions has appeared on the generated green light. Hence, the studied glasses possessed many properties qualifies them for optoelectronics and nonlinear optics applications.

## Data Availability

Data will be made available on request. The datasets generated during and/or analysed during the current study are available from the corresponding author on reasonable request.
